# High-Density GBS-Based Genetic Linkage Map Construction and QTL Identification Associated With Yellow Mosaic Disease Resistance in Bitter Gourd (*Momordica charantia* L.)

**DOI:** 10.3389/fpls.2021.671620

**Published:** 2021-06-24

**Authors:** Gurpreet Kaur, Mamta Pathak, Deepak Singla, Abhishek Sharma, Parveen Chhuneja, Navraj Kaur Sarao

**Affiliations:** ^1^School of Agricultural Biotechnology, Punjab Agricultural University, Ludhiana, India; ^2^Department of Vegetable Science, Punjab Agricultural University, Ludhiana, India

**Keywords:** yellow mosaic disease, bitter gourd, genotyping by sequencing, linkage map, quantitative trait loci

## Abstract

Yellow mosaic disease (YMD) in bitter gourd (*Momordica charantia*) is a devastating disease that seriously affects its yield. Although there is currently no effective method to control the disease, breeding of resistant varieties is the most effective and economic option. Moreover, quantitative trait locus (QTL) associated with resistance to YMD has not yet been reported. With the objective of mapping YMD resistance in bitter gourd, the susceptible parent “Punjab-14” and the resistant parent “PAUBG-6” were crossed to obtain F_4_ mapping population comprising 101 individuals. In the present study, the genotyping by sequencing (GBS) approach was used to develop the genetic linkage map. The map contained 3,144 single nucleotide polymorphism (SNP) markers, consisted of 15 linkage groups, and it spanned 2415.2 cM with an average marker distance of 0.7 cM. By adopting the artificial and field inoculation techniques, F_4:5_ individuals were phenotyped for disease resistance in Nethouse (2019), Rainy (2019), and Spring season (2020). The QTL analysis using the genetic map and phenotyping data identified three QTLs *qYMD.pau_3.1*, *qYMD.pau_4.1*, and *qYMD.pau_5.1* on chromosome 3, 4, and 5 respectively. Among these, *qYMD.pau_3.1*, *qYMD.pau_4.1* QTLs were identified during the rainy season, explaining the 13.5 and 21.6% phenotypic variance respectively, whereas, during the spring season, *qYMD.pau_4.1* and *qYMD.pau_5.1* QTLs were observed with 17.5 and 22.1% phenotypic variance respectively. Only one QTL *qYMD.pau_5.1* was identified for disease resistance under nethouse conditions with 15.6% phenotypic variance. To our knowledge, this is the first report on the identification of QTLs associated with YMD resistance in bitter gourd using SNP markers. The information generated in this study is very useful in the future for fine-mapping and marker-assisted selection for disease resistance.

## Introduction

Bitter gourd (*Momordica charantia* L.) is also popularly known as bitter melon, bitter cucumber, balsam pear, karela, and African pear (Basch et al., 2003). It belongs to the family *Cucurbitaceae* and genus *Momordica* having chromosome number 2*x* = 2*n* = 22 and is one of the important vegetable crops grown in the tropics. There is uncertainty in the exact place of the origin of bitter gourd but according to various molecular studies, east India is supposed as its center of origin, whereas China is its secondary center of diversity (Dey et al., 2006; Gaikwad et al., 2008). Among various bitter gourd growing regions, India, Malaysia, China, South America, and Africa are on the top of the list (Miniraj et al., 1993). This crop is mainly cultivated in warm climates, so the summer season is the most appropriate season for its production in India. Though all parts of the plant are edible, the immature fruits are consumed most commonly. Bitter gourd is rich in vitamins and nutrients (Raman and Lau, 1996; Blum et al., 2012) and possess antioxidant (Krishnaiah et al., 2011) and anti-hepatotoxic (Semiz and Sen, 2007) and anti-cancer properties (Brennan et al., 2012).

Due to these medicinal properties, people have shown interest in including *M. charantia* in their diet, and hence the demand for bitter gourd has increased (Warrier et al., 1997). But its production is decreasing due to various diseases caused by different pathogens like bacteria, fungi, and viruses. Among these, yellow mosaic disease (YMD) has proven to be the most destructive viral disease which could result in up to 100% yield loss depending upon the developmental stage of the crop (Giri and Mishra, 1986). Various efforts were made to study the transmission, causal pathogen, host range, and etiological impact of the disease. YMD is caused by bipartite geminivirus belonging to genus *Begomovirus* and family *Geminiviridae*, having circular topology and a unique single-stranded DNA genome. This group of viruses can easily manipulate their genome which helps them to infect a wide range of dicotyledonous plants. Begomoviruses also cause YMD in some other crops like cotton, mungbean, soybean, cucumber, okra, etc. The viruses affect the host at all growth stages and are transmitted by *Bemisia tabaci* (whitefly). The disease symptoms include typical mosaic, upward leaf curling, mottling, crinkling, and severe stunting symptoms (Matthew et al., 1991).

To date, many viruses related to YMD have been identified and characterized. These include *Papaya ring spot virus* (Gonzalez et al., 2003), *Zucchini yellow mosaic virus* (ZYMV) (Fukumotu et al., 1993), *Water melon silver mottle virus* (Tokashiki and Yasuda, 1991), *Water melon mosaic virus-1* (Tomar and Jitendra, 2005), *Bitter gourd yellow mosaic virus* (Rajinimala et al., 2005), *Tomato leaf curl distortion virus* (Tahir and Haider, 2005), *Cucumber mosaic virus* (Takami et al., 2006), and *Indian cassava mosaic virus* (Rajinimala and Rabindran, 2007). Recently, *Squash vein yellowing virus* and *Cucurbit leaf crumple virus* (Adkin et al., 2008), *Melon yellow spot virus* (Takeuchi et al., 2009) and *Pepper leaf curl Bangladesh virus* (Raj et al., 2010) have also been investigated on bitter gourd. Association of *Tomato leaf curl New Delhi virus* with YMD of bitter gourd from India has been reported in isolation (Tiwari et al., 2010) or as a mixed infection with ZYMV by Sharma et al. (2015) while Ali et al. (2010) reported the presence of *Tomato leaf curl Palampur virus* in YMD affected bitter gourd plants. The simultaneous occurrence of different viruses in bitter gourd plants results in the mosaic complex. The ability of genome manipulation by recombination coupled with vector transmission of viruses by vectors to new areas has doubled the impact of the diseases (Padidam et al., 1999).

The chemical methods used to control the vector whitefly population generally pollute the environment and cause detrimental health issues. One of the most sustainable ways of managing the YMD in bitter gourd is through host plant resistance. So the development of improved disease-resistant cultivars should be the primary goal for the breeders but the major problem in YMD resistant breeding is the screening for the disease. The development of non-uniform disease due to the fluctuation in vector population is generally observed in different locations and seasons. This problem can be solved by Marker-assisted selection (MAS), as markers linked to traits of interest help to reduce the number, time, and cost of phenotypic evaluations. So far, there is no report on mapping and DNA markers associated with YMD resistance in bitter gourd.

An enriched genetic linkage map is essential to identify the location of genes controlling disease resistance. A few linkage maps have been constructed in bitter gourd by using DNA molecular markers. Kole et al. (2012) developed the first linkage map using amplified fragment length polymorphism (AFLP) markers. Simple sequence repeats (SSR), AFLP, and sequence-related amplified polymorphism (SRAP) markers were used in the linkage map developed by Wang and Xiang (2013). Owing to the development made in next-generation sequencing technologies, sequencing-based genotyping methods like Restriction enzyme-associated DNA sequencing (RAD-seq; Baird et al., 2008) and genotyping by sequencing (GBS; Elshire et al., 2011) can be used in single nucleotide polymorphism (SNP) markers discovery, linkage map development and quantitative trait locus (QTL) identification in bitter gourd. Urasaki et al. (2017) published the first short-read scaffold-level assembly of bitter gourd inbred line, OHB3-1, and constructed a linkage map consisting of 11 linkage groups using RAD-seq analysis. Cui et al. (2018) also used RAD-seq SNP markers for linkage map construction in bitter gourd and GBS without reference genome was used by Rao et al. (2018). Recently, a chromosome-level genome assembly is published along with a linkage map, and it is currently one of the most complete assemblies among publicly available genomes in cucurbitaceae (Matsumura et al., 2020).

In this study, we used a reference-based GBS approach for SNP identification and genotyping of a mapping population, segregating for YMD. The identified SNPs were used in the construction of linkage maps and detection of loci associated with YMD.

## Materials and Methods

### Plant Material

Resistant parent, PAUBG-6 and susceptible parent Punjab-14 were used for the development of the mapping population. The Department of Vegetable Science, Punjab Agricultural University (PAU), screened bitter gourd germplasm against YMD following field screening technique and identified bitter gourd genotypes with YMD resistance. Of these PAUBG-6 was found to be a stable source of resistance to this viral disease. Whereas the recipient Punjab-14 is an elite cultivar with green shining fruits suitable for cooking through stuffing. Female flowers of Punjab-14 were hand-pollinated with pollen from male flowers of PAUBG-6 to generate F_1_ plants. F_1_s were further hand self-pollinated to produce F_2_ seeds and individual F_2_ plants were planted again. A total of 101 F_2_ plants were selfed individually to produce F_2:3_ lines. These lines were selfed in subsequent generations to develop F_2:4_ and F_2:5_ lines (Supplementary Figure 1). The populations were grown according to the standard agronomic practices in the spring and rainy seasons of the years 2017–2020.

### Phenotypic Screening and Disease Evaluation

F_4:5_ individuals were phenotyped for YMD resistance. The F_4:5_ individuals were screened using both artificial and natural inoculations to achieve a uniform vector distribution. A total of 101 F_4:5_ families were subdivided into three subsets, which were then grown in three different environments consisting of three replications in randomized block design. The first subset was grown in a nethouse (2019), the second during the rainy season (2019), and the third during the spring season (2020) to study the disease response. For artificial inoculation, the non-viruliferous whiteflies (*B. tabaci*) were reared on virus-free cotton plants in an insect-proof cage of fine mesh held in a controlled temperature chamber at 28°C with a photoperiod of 14 h and 30–50% relative humidity (Polston and Capobianco, 2013). The virus-infected bitter gourd plants showing YMD symptoms were collected from the field and were used as a primary source of the virus. The non-viruliferous whiteflies were collected from cotton plants and fed on virus-infected bitter gourd plants for virus acquisition for 24 h. Later these viruliferous whiteflies were released on test plants sown in plug trays and maintained in the same cage. The F_4:5_ inoculated individuals were then transplanted in testing environments for screening. The spreader row technique was adopted in screening to evaluate lines against YMD infection. About 10 F_5_ seedlings from each F_4_ plant were planted on well-prepared hills of 1.5 m wide beds with a plant-to-plant distance of 45 cm. Punjab-14 was planted as indicator-cum-infestor row after every four test rows. The main source of virus transmission is the whitefly; to maintain the natural population of whiteflies in the experimental field, insecticide spray was avoided. In field conditions during the rainy and spring seasons, plants were under constant disease pressure while the plants in the nethouse were exposed to viruliferous whiteflies only once before transplanting. The disease reaction was recorded at the time of the first harvest using a grading scale suggested by Arunachalam et al. (2002). The lines with a score of 0 were considered as highly resistant, a score of 1 as resistant, a score of 2 as moderately resistant, a score of 3 as moderately susceptible, a score of 4 as susceptible, and a score of 5 as highly susceptible (Supplementary Figure 2). The percent disease index (PDI) was calculated for each line by applying the formula given by McKinney (1923):

PDI=Σ[(s×n)/(S×N)]×100%

Here, *s*: disease grade; *n*: number of plants in the disease grade, *S*: highest disease rating scale, and *N*: total numbers of plants.

### DNA Isolation, Library Construction, and Sequencing

Genomic DNA was isolated from fresh leaf tissue of F_4_ plants as well as from parents for genotyping by using CTAB (Cetyl Trimethyl Ammonium Bromide) method as given by Doyle and Doyle (1990). The DNA was quantified using NanoDrop^TM^ 1000 spectrophotometer (Thermo Scientific, Wilmington, United States) whereas, the integrity and quality of DNA was checked on 0.8% agarose gel. High-quality DNA diluted to ∼100 ng/μl was used for GBS analysis. DNA digestion, adapter ligation, library construction, and sequencing were carried out by Novogene (China). For bitter gourd library preparation, two restriction enzymes *Mse1* and *Msp1* were used. The library was sequenced with the paired-end (PE) read length of 150 bp using Illumina HiSeq 2000.

### Single Nucleotide Polymorphism Identification and Genotyping

The PE sequence reads from FASTQ files were filtered based on criteria for sequence quality and length using a trimmomatic tool (Bolger et al., 2014). Sequence reads having < 50 bp of length were removed. The filtered reads were aligned to the reference genome of genotype sequence OHB3-1 of bitter gourd using Bowtie 2 software (Langmead and Salzberg, 2012) with default parameters. Sequence alignment map (SAM) to binary alignment map (BAM) format conversion followed by sorting was done using SAMtools. SNP calling was performed using the BCFtools “*mpileup*” command. The resulting SNPs were filtered out with 25% missing data, minimum read depth 4, minimum base quality 20, and minor allele frequency (MAF) of ≤0.05. Then, further filters like SNPs with any missing site in one or both parents and any site with a heterozygous call at one or both parents were also removed. The monomorphic SNP sites were rejected and only polymorphic SNP sites called in both the parents were selected. A total of 4196 SNPs with contrasting alleles were retained after converting the generated SNPs into a parent-based format and were further used for linkage map construction.

### Genetic Linkage Map Construction

The filtered SNPs from the 101 lines were used for the construction of the linkage map using Mapdisto version 1.7.7 (Lorieux, 2012). The chi-square test (χ^2^) was performed to check the segregation of each SNP marker for any deviation from the expected Mendelian ratio. Highly distorted and unlinked markers were excluded from the analysis. The SNPs were split into linkage groups with a recombination frequency of 0.3 at a logarithm of odds (LOD) value of 3–6. The markers were ordered and rippled by the Seriation method and SARF (Sum of Adjacent Recombination Frequencies) criteria respectively. Finally, the Kosambi mapping function was applied to compute map distance (cM). Linkage groups (LGs) were drawn using MapChart V2.2 (Voorrips, 2002).

### Quantitative Trait Locus Analysis

The QTL analysis was conducted using the IciMapping software program (Meng et al., 2015) by ICIM-ADD (Inclusive composite interval mapping) method. Only QTLs having LOD values > 3.0 and phenotypic variance (PV) of more than 10% were considered putative QTLs for YMD resistance. Nomenclature for the QTL was denoted as, for instance, qYMD_pau4.1 where “qYMD” represents the QTL for resistance to YMD, “pau” represents institution name, and 4_1 represents the first QTL on chromosome 4.

## Results

### Phenotypic Evaluation of Mapping Population

**FIGURE 1:**
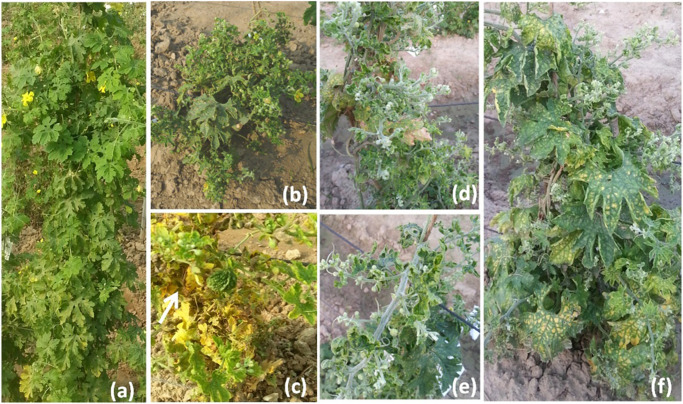
Yellow mosaic disease symptom variations in field **(a)** normal plant, **(b)** stunted growth, **(c)** small deformed fruits, **(d)** shortening of internodes, **(e)** stem hairiness, and **(f)** reduction in the number of flowers.

**FIGURE 2:**
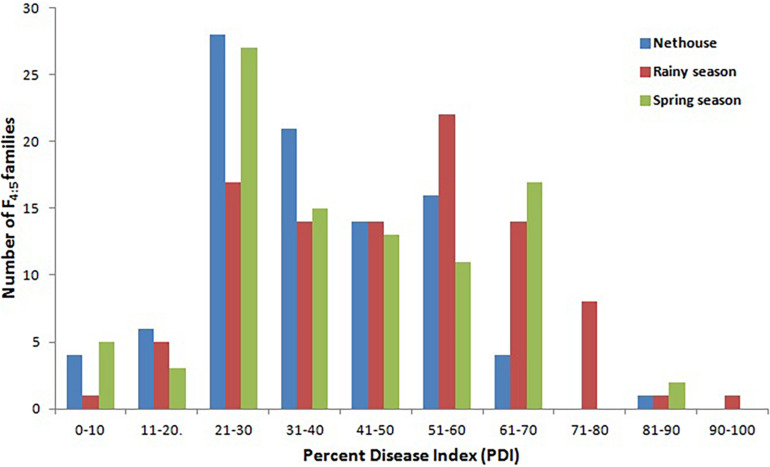
Frequency distribution for YMD reaction in F_4:5_ families derived from the cross between Punjab-14 and PAUBG-6 in three environments.

The disease development was very good in three environments. The YMD symptoms like stunted growth, shortening of internodes, deformed fruits, and increase in hairiness were observed in the experimental field (Figure 1). The parents of the mapping population showed contrasting phenotypic reactions for YMD resistance. In F_1_ all plants were susceptible indicating disease resistance to be recessive. In the case of the F_4:5_ population, the mean PDI score ranged from 6 to 82, 9 to 98, and 6 to 98 with a coefficient of variation (CV) of 42.1, 38.9, and 44.7% for the nethouse, rainy, and spring seasons respectively (Table 1). The frequency distributions (Figure 2) and the absolute value of <1 for skewness and kurtosis (Table 1) for the trait demonstrate the continuous and normal distribution of the disease reaction data.

**TABLE 1:** Descriptive statistical result for F_4:5_ population derived from the cross of Punjab-14 and PAUBG-6.

Environment	Skewness	Kurtosis	Percent disease index		Range	Mean	Coefficient of variance (CV%)
			Min	Max			
Nethouse	0.300	−0.294	6	82.0	760	36.5	42.1
Rainy season	0.095	−0.602	9	98.0	89.0	47.3	38.9
Spring season	0.171	−0.507	6	98.0	92.0	42.9	44.7

### High-Throughput Sequencing and SNP Identification

**FIGURE 3:**
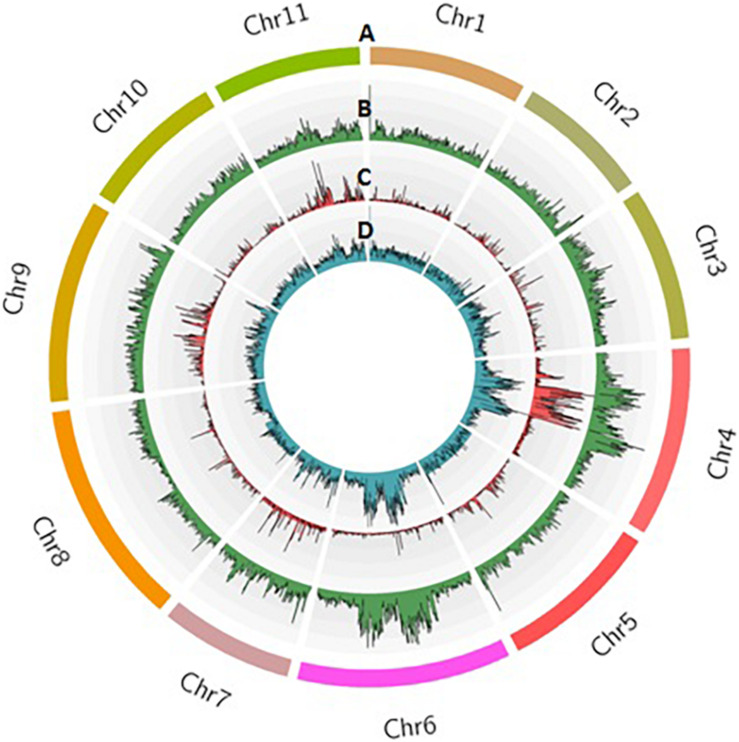
Distribution of SNPs identified in the F_4_ population from Punjab-14 x PAUBG-6 in bitter gourd. Track A represents 11 chromosomes in different colors. Tracks B, C, and D represent raw SNPs, filtered SNPs, and mapped SNPs within 100 kb window on 11 chromosomes.

Sequencing of 101 F_4_ lines generated over 144 million PE reads (21.73 GB) that had a length of 150 bp. In parents, a total of 1.8 and 1.5 million reads in Punjab-14 and PAUBG-6 were generated respectively. The reads from F_4_ individuals ranged from 0.5 to 3.3 million reads with an average of 1.4 million reads (Supplementary Figure 3). Then, the cleaned 88–99% sequencing reads were successfully aligned with a reference genome (DNA Data Bank of Japan, accession nos. BLBB01000001–BLBB01000011) for SNP identification. As a result, 4,31,148 raw SNPs were identified, across the 11 chromosomes. Out of these, 1, 54,552 SNPs were polymorphic with a polymorphism rate of 35.84%. The 17,488 SNPs were filtered with filtering 5% MAF and 25% missing data. These SNPs were then filtered for Chi-Square at *p* = 0.001 which resulted in 4196 SNPs (Table 2). These 4196 SNPs were then used for linkage map construction. The distribution of SNPs on 11 chromosomes is illustrated in Figure 3.

**TABLE 2:** Detail of SNP sites identified in chromosomes of bitter gourd before and after filtering.

Chromosome	No. of SNPs identified	Polymorphic SNPs	No. of SNPs filtered
1	28,880	11,285	273
2	26,078	9,172	124
3	35,221	12,557	346
4	62,830	18,616	390
5	30,768	12,230	366
6	75,528	24,684	191
7	31,657	11,557	313
8	38,277	14,411	443
9	42,111	16,628	1,037
10	28,516	11,023	197
11	31,282	12,389	516
Total	4,31,148	1,54,552	4,196

### Single Nucleotide Polymorphism Based High-Density Linkage Maps

**FIGURE 4:**
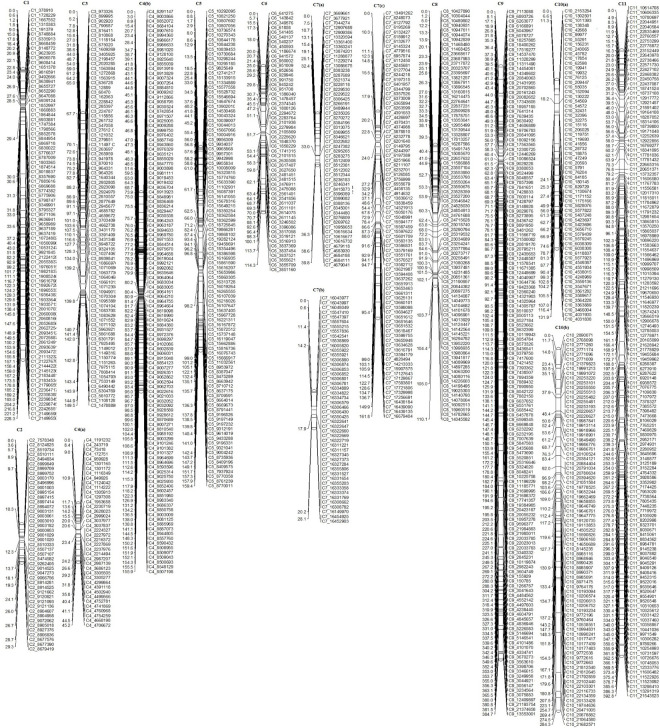
Genetic linkage map developed from the F_4_ population derived from a cross between Punjab-14 and PAUBG-6.

Out of 4,196 SNPs, 3,144 formed 15 linkage groups spanning a cumulative length of 2415.2 cM (Figure 4). Linkage groups were named according to chromosomes. The number of linkage groups obtained was more than the haploid number of chromosomes in the bitter gourd genome. This was due to insufficient linkage among SNP loci on the linkage group corresponding to chromosome 4, 7, and 10, causing the chromosome to split into more than one groups which were named as chromosome 4(a), 4(b), 7(a), 7(b), 7(c), 10(a), and 10(b). Overall, the linkage map contained 209.6 SNPs per linkage group with an average length of 161.01 cM and a mean marker interval of 0.7 cM. The number of mapped SNPs per linkage group varied from 40 on chromosome 4(a) to 957 on chromosome 9. The smallest linkage group was chromosome 2, which contained 45 SNPs spanning a length of 29.3 cM. The largest linkage group was chromosome 11, which had 423 SNPs and a length of 392.8 cM. The maximum gap size in the linkage map was 31.6 cM on chromosome10(c) (Table 3).

**TABLE 3:** Summary statistics of the bitter gourd genetic linkage map constructed using F_4_ individuals derived from the cross of Punjab-14 and PAUBG-6.

LG	No. of markers	Length (cM)	Av. spacing (cM)	Max. spacing (cM)
1	195	228.3	1.2	30.4
2	45	29.3	0.7	4.7
3	232	149.5	0.6	11.2
4	40	45.2	1.2	8.4
5	238	155.9	0.7	7.9
6	282	159.4	0.6	17.0
7	80	114.3	1.4	17.3
8	54	39.8	0.8	7.5
9	142	161.8	1.1	13.3
10	48	28.1	0.6	10.7
11	259	110.1	0.4	10.7
12	957	384.7	0.4	17.6
13	66	131.8	2.0	22.5
14	83	284.3	3.5	31.6
15	423	392.8	0.9	16.1
Overall	3,144	2,415.2	0.8	31.6

### Detection of QTLs Conferring YMD Resistance

**FIGURE 5:**
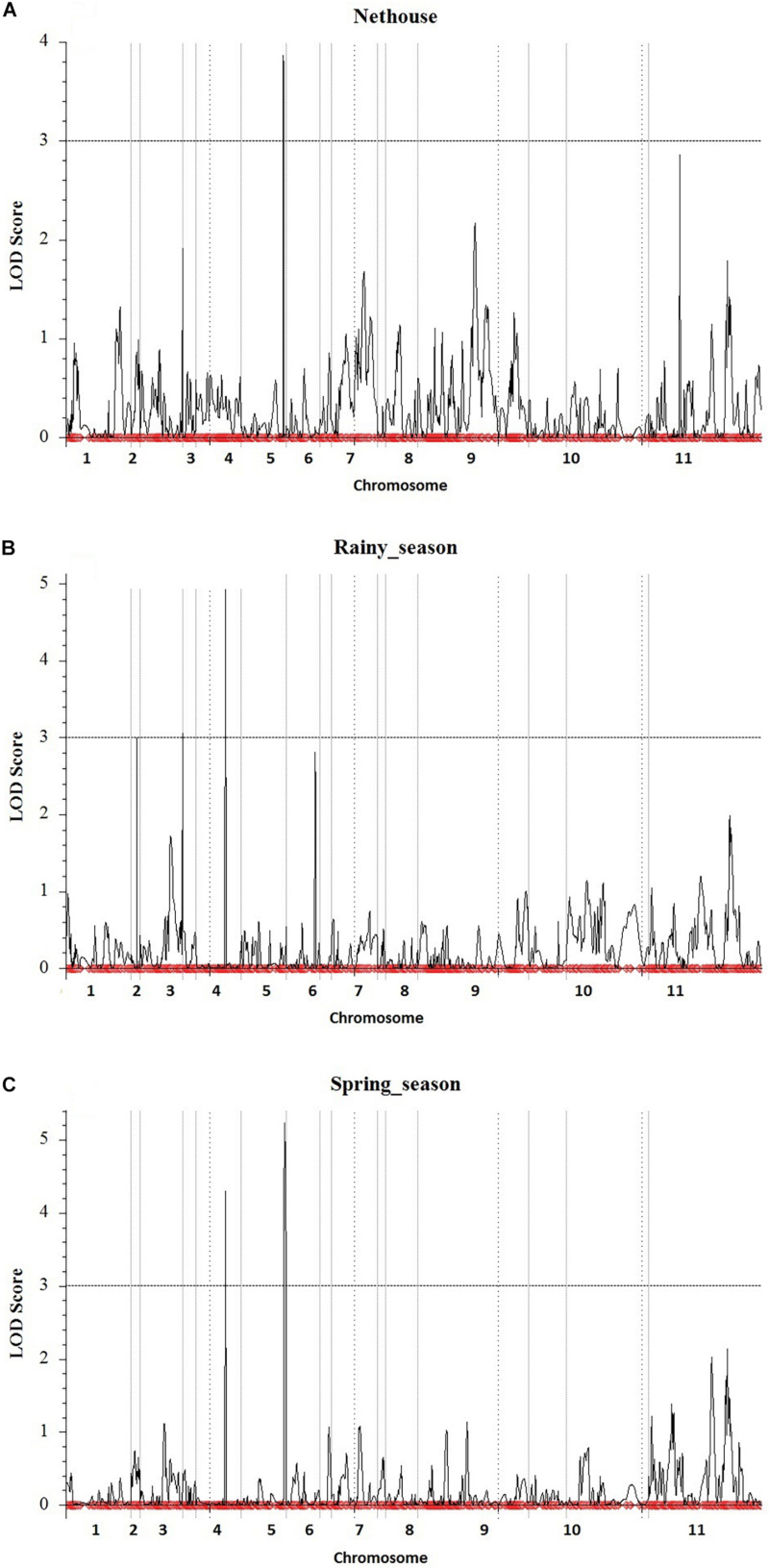
Quantitative trait locus detected for yellow mosaic disease resistance in the **(A)** Nethouse, **(B)** Rainy season of the year 2019, and **(C)** Spring season of the year 2020. Plots indicate the genetic coordinate on the *x*-axis and LOD score on the *y*-axis of detected QTLs.

The linkage map developed from genotyping of 3,144 SNP markers was integrated with phenotypic disease reaction data from three independent trails and was analyzed using the ICIM method. Three QTLs for YMD resistance were identified on chromosome 3, 4, and 5 and designated as *qYMD_pau3.1*, *qYMD_pau.4.1*, and *qYMD_pau.5.1* respectively (Table 4). The QTL *qYMD_pau3.1* was identified for the rainy season at position 149 cM between flanking SNP markers C3_11081069 and C3_14788898, showing an LOD score of 3.05 (Figure 5). This QTL explained 13.5% phenotypic variance (PV) with an additive effect of 5.60. The QTL *qYMD_pau.4.1* flanked by C4_8810605-C4_8985192 markers was detected in the rainy season at 104 cM with an LOD score of 4.97 and in the spring season with an LOD score of 4.29 on chromosome 4. This QTL showed an additive effect of −6.82 and accounted for 17.5–21.6% disease score variation for both seasons. The QTL *qYMD_pau.5.1* was identified in the spring season with a high LOD score of 5.23 and 22.1% phenotypic variance at position 152 cM on chromosome 5 with flanking markers C5_7937924-C5_8770358 whereas for the nethouse QTL was detected on chromosome 5 at position 148 cM flanked by C5_8409196-C5_8409575 with LOD value of 3.87 and explained 15.6% phenotypic variance. This QTL lies within the same confidence interval as the QTL region identified in the spring season on chromosome 5.

**TABLE 4:** Quantitative trait locus identified for yellow mosaic disease resistance in bitter gourd.

Environment	QTL	Chromosome	Position	LOD	PVE (%)	Add	Marker interval	Confidence interval (cM)
Nethouse	*qYMD_pau5.1*	5	148	3.86	15.6	−6.32	C5_8409196-C5_8409575	147.5–149.5
Rainy season	*qYMD_pau3.1*	3	149	3.05	13.5	5.60	C3_11081069-C3_14788898	147.5–149.0
	*qYMD_pau4.1*	4	104	4.97	21.6	−6.82	C4_8810605-C4_8985192	101.5–104.5
Spring season	*qYMD_pau4.1*	4	104	4.29	17.5	−6.79	C4_8810605-C4_8985192	101.5–104.5
	*qYMD_pau5.1*	5	152	5.23	22.1	−7.83	C5_7937924-C5_8770358	149.5–154.5

*LOD – logarithm of odds, PVE – phenotypic variance explained, Add – additive effect.*

## Discussion

Yellow mosaic is one of the severe threats in bitter gourd production that needs to be controlled by using a sustainable, environment-friendly approach. Breeding of resistant varieties through MAS is the most effective way as it has proved its importance in achieving desirable phenotypic variation in less time. There is no report of inheritance and mapping of YMD resistance gene in bitter gourd to date and hence no marker linked to the trait has been reported. Therefore, this study aimed at identifying YMD resistance genes through the GBS approach. GBS generates a large number of SNPs, which are used in the construction of high-density linkage maps and gene tagging. This approach has been used in cucurbits like watermelon (Branham et al., 2016; Meru and McGregor, 2016), bitter gourd (Rao et al., 2018), bottle gourd (Wu et al., 2017), squash (Zhang et al., 2015), musk melon (Chang et al., 2017; Daley et al., 2017; Branham et al., 2018), and pumpkin (Montero-Pau et al., 2017) for construction of linkage map and QTL mapping. Biparental mapping in integration with SNP genotyping is becoming a powerful tool for dissecting complex traits in plants (Xu et al., 2017). In this regard, a genetic map with reasonable marker density and genetic regions associated with YMD resistance identified in the present study is an important milestone in bitter gourd breeding.

We used GBS to discover and genotype SNPs in F_4_ mapping populations, derived from the cross between Punjab-14 (susceptible parent) and PAUBG-6 (resistant parent). Sequencing of mapping population resulted in the identification of 144 million reads in the F_4_ population with an average of 1.4 million reads per individual. The 88–99% alignment rate with the reference genome indicates the sequencing accuracy. We used chromosome level genome assembly (Matsumura et al., 2020) as a reference to find SNPs on bitter gourd chromosomes while in previous reports either the short reads scaffold assembly (Cui et al., 2018) or non-reference genome (Rao et al., 2018) approach was used for SNP discovery. About 4,31,148 raw SNPs were identified which were passed through stringent filter criteria like missing percentage, MAF, and segregation behavior to select SNPs for constructing high-density genetic maps. This resulted in the reduction of SNPs from millions to few thousands, however, this is very common in GBS data, and similar results have been reported in many other GBS-based genetic mapping studies (Zhang et al., 2015; Mathivathana et al., 2019). In summary, in the present study, a total of 3,144 SNPs were finally mapped on the linkage map. The number of mapped markers was low in comparison to the identified SNPs between parental lines may be due to the limited sequencing depth used for GBS. The linkage map spanning a total 2415.2 cM length consisted of 15 linkage groups with a mean marker interval of 0.7 cM an average of 209.6 markers for each linkage group. The mean marker density (marker/cM) 1.30 is higher than 0.86 reported by Rao et al. (2018), 0.30 by Matsumura et al. (2014), 0.42 by Urasaki et al. (2017), and 0.46 by Cui et al. (2018). The linkage map produced in this study resulted in more linkage groups than haploid chromosome number (*n* = 11). This may be due to the absence of linkage between SNPs in that region due to a relatively small F_4_ population size. The population development in bitter gourd crops is laborious and time-consuming, as the crop is highly cross-pollinated and monoecious in nature. But efforts were made to advance the population from F_2_ to F_2:4_ and F_2:5_ lines to achieve more homozygosity and accurate results.

Infection at the early developmental stage results in 100% loss; to avoid this situation, phenotyping for the disease resistance was performed in the F_4:5_ population. The virus incidence and disease severity vary with varying seasons and environments in India. The temperature during the spring season is generally high with low humidity as compared to the high humidity of the rainy season, which helps the vector population to grow at a faster rate. This results in increasing the whitefly population and virus incidence. Mapping populations in the present study were phenotyped in three independent environment conditions. In the nethouse, the plants were challenged with viruliferous whiteflies for once before transplanting, while during the rainy season (2019) and spring season (2020) the plants were under constant disease pressure. Generally, seasonal and annual variations in the field influence the virus incidence and severity of the disease, so to minimize these variations, artificial inoculations are done before transplanting the test material in fields for further screening (Picó et al., 1998). So to avoid the non-uniform development of disease while screening, artificial and natural whitefly mediated transmission techniques were used. The phenotypic data depicted the polygenic inheritance of the disease. Huge variations were observed among progenies across the test environments which can be seen in mean PDI scores (Table 1). The phenotypic variation ultimately affects the identification of stable QTLs. This might be due to the presence of environment interactions/pathogenic variability across the seasons.

Quantitative trait locus mapping for YMD resistance has revealed three significant QTLs *qYMD_pau3.1*, *qYMD_pau.4.1*, and *qYMD_pau.5.1* having major effects with PV of more than 10% on chromosomes 3, 4, and 5. The identified QTLs in the present study are novel as this is the first report of mapping QTLs for YMD resistance in bitter gourd. Out of these, QTL *qYMD_pau.4.1* was identified on chromosome 4 in two seasons. The QTL *qYMD_pau3.1* is observed during the rainy season, while *qYMD_pau5.1* is observed during the spring season and under nethouse conditions. The additive effect of *qYMD_pau4.1* and *qYMD_pau5.1* indicates that the alleles for resistance are contributed by the resistant parent (PAUBG-6) while the alleles for the resistance due to *qYMD_pau3.1* are contributed by the susceptible parent (Punjab-14). Altogether, these results suggested that the dense genetic map provides accurate detection of QTLs for YMD resistance in bitter gourd. The inclusive composite interval mapping (ICIM-ADD) method was used in the identification of QTLs, as it has increased detection power, produces a lower false detection rate, and avoids the possible sampling variance and the complicated background marker selection process while retaining all the advantages of CIM.

## Conclusion

In this study, SNPs were identified from the bitter gourd genome by using the GBS approach. Using the identified SNPs a linkage map was developed between Punjab-14 and PAUBG-6. Three QTLs for YMD resistance have been mapped successfully, and this is the first report of mapping YMD resistance in bitter gourd to the best of our knowledge. The information generated on QTLs is very useful for its fine-mapping and for MAS for the trait in the future. These can be applied for the development of YMD resistant genotypes and molecular dissection of YMD resistance in bitter gourd.

## Data Availability Statement

The datasets presented in this study can be found in online repositories. The names of the repository/repositories and accession number(s) can be found below: https://www.ncbi.nlm.nih.gov/, PRJNA702876.

## Author Contributions

GK performed the YMD phenotyping, contributed to the analysis of SNP data, linkage map construction, and QTL analysis. AS contributed to the YMD phenotyping and data analysis. DS contributed to the SNP identification. NS and MP designed and supervised the project. NS edited the manuscript. PC gave critical suggestions throughout the study and also edited the manuscript. All authors contributed to the article and approved the submitted version.

## Conflict of Interest

The authors declare that the research was conducted in the absence of any commercial or financial relationships that could be construed as a potential conflict of interest.

## References

[B1] Adkin S. Webb S. E. Baker C. A. Kousik C. S. (2008). Squash vein yellowing virus detection using nested PCR demonstrates that the cucurbit weed *M. charantia* is a reservoir host. *Plant Dis.* 92 1119–1123. 10.1094/pdis-92-7-1119 30769530

[B2] Ali I. Malik A. H. Mansoor S. (2010). First report of Tomato leaf curl Palampur virus on bitter gourd in Pakistan. *Dis. Notes* 94:276. 10.1094/PDIS-94-2-0276A 30754274

[B3] Arunachalam P. Radhakrishnan V. Mathew S. Kumar S. (2002). Reaction of bitter gourd genotypes against distortion mosaic virus. *Veg. Sci.* 29 55–57.

[B4] Baird N. A. Etter P. D. Atwood T. S. Currey M. C. Shiver A. L. Lewis Z. A. (2008). Rapid SNP discovery and genetic mapping using sequenced RAD markers. *PLoS One* 3:e3376. 10.1371/journal.pone.0003376 18852878 PMC2557064

[B5] Basch E. Garbardi S. Ulbricht C. (2003). Bitter melon (*Momordica charantia*): a review of efficacy and safety. *Am. J. Health Syst. Phram.* 60 356–359. 10.1093/ajhp/60.4.356 12625217

[B6] Blum A. Loerz C. Martin H. J. Claudia A. Staab-Weijnitz C. A. Maser E. (2012). *Momordica charantia* extract, a herbal remedy for type-2 diabetes, contains a specific 11β-hydroxysteroid dehydrogenase typr 1 inhibitor. *J. Steroid. Biochem. Mol. Biol.* 128 51–55. 10.1016/j.jsbmb.2011.09.003 22001161

[B7] Bolger A. M. Lohse M. Usade B. (2014). Trimmomatic: a flexible trimmer for Illumina sequence data. *Bioinformatics* 30 2114–2120. 10.1093/bioinformatics/btu170 24695404 PMC4103590

[B8] Branham S. E. Levi A. Farnham M. W. Wechter W. P. (2016). A GBS-SNP-based linkage map and quantitative trait loci (QTL) associated with resistance to *Fusarium oxysporum* f. sp. niveum race 2 identified in *Citrullus lanatus* var. *citroides*. *Theor. Appl. Genet.* 130 319–330. 10.1007/s00122-016-2813-0 27803951

[B9] Branham S. E. Levi A. Katawczik M. Fei Z. Wechter W. P. (2018). Construction of a genome-anchored, high-density genetic map for melon (*Cucumis melo L*.) and identification of *Fusarium oxysporum* f. sp. *melonis* race 1 resistance QTL. *Theor. Appl. Genet.* 131 829–837. 10.1007/s00122-017-3039-5 29372283

[B10] Brennan V. C. Wang C. M. Yang W. H. (2012). Bitter melon (*Momordica charantia*) extract suppresses adrenocortical cancer cell proliferation through modulation of the apoptotic pathway, steroidogenesis and insulin like growth factor type 1 receptor/RAC-α serine/threonine-protein kinase signaling. *J. Med. Food* 15 325–334. 10.1089/jmf.2011.0158 22191569

[B11] Chang C. W. Wang Y. H. Tung C. W. (2017). Genome-wide single nucleotide polymorphism discovery and the construction of a high-density genetic map for melon (*Cucumis melo L*.) using genotyping-by-sequencing. *Front. Plant Sci.* 8:125. 10.3389/fpls.2017.00125 28220139 PMC5292975

[B12] Cui J. Luo S. Niu Y. Huang R. Wen Q. Su J. (2018). A RAD-based genetic map for anchoring scaffold sequences and identifying QTLs in bitter gourd (*Momordica charantia*). *Front. Plant Sci.* 9:477. 10.3389/fpls.2018.00477 29706980 PMC5906717

[B13] Daley J. Branham S. Levi A. Hassell R. Wechter P. (2017). Mapping resistance to *Alternaria cucumerina* in *Cucumis melo*. *Phytopathology* 107 427–432. 10.1094/PHYO-06-16-0246-R 27868479

[B14] Dey S. S. Singh A. K. Chandel D. Behera T. K. (2006). Genetic diversity of bitter gourd (*Momordica charantia* L.) genotypes revealed by RAPD markers and agronomic traits. *Sci. Hortic.* 109 21–28. 10.1016/j.scienta.2006.03.006

[B15] Doyle J. J. Doyle J. L. (1990). Isolation of plant DNA from fresh tissue. *Focus* 12 13–15.

[B16] Elshire R. J. Glaubitz J. C. Sun Q. Poland J. A. Kawamoto K. Buckler E. S. (2011). A robust, simple genotyping-by-sequencing (GBS) approach for high diversity species. *PLoS One* 6:e19379. 10.1371/journal.pone.0019379 21573248 PMC3087801

[B17] Fukumotu F. Terami F. Ishmi M. (1993). Zucchini yellow mosaic virus isolated from wax gourd and balsam pear (*M*. *charantia*). *Proc. Kanto Tosan Plant Prot. Soc.* 40 101–103.

[B18] Gaikwad A. B. Behera T. K. Singh A. K. Chandel D. Karihaloo J. L. Staub J. E. (2008). AFLP analysis provides strategies for improvement of bitter gourd (*Momordica charantia*). *Hort. Sci.* 43 127–133. 10.21273/hortsci.43.1.127

[B19] Giri B. K. Mishra M. D. (1986). *National seminar on whitefly-transmitted plant virus diseases.* New Delhi: Indian Agricultural Research Institute, 22.

[B20] Gonzalez V. A. Trujillo P. G. Vegas G. A. (2003). Use of differential hosts to identify strains of *Papaya ring spot virus*. *Rev. Mex. Fitopatol.* 21 67–70.

[B21] Kole C. Olukolu B. A. Kole P. Rao V. K. Bajpai A. Backiyarani S. (2012). The first genetic map and positions of major fruit trait loci of bitter melon (*Momordica charantia*). *J. Plant. Sci. Mol. Breed.* 1 1–10. 10.7243/2050-2389-1-1

[B22] Krishnaiah D. Sarbatly R. Nithyanandam R. (2011). A review of the antioxidant potential of medicinal plant species. *Food Bioprod. Process.* 89 217–233. 10.1016/j.fbp.2010.04.008

[B23] Langmead B. Salzberg S. (2012). Fast gapped-read alignment with Bowtie 2. *Nat. Methods* 9 357–359. 10.1038/nmeth.1923 22388286 PMC3322381

[B24] Lorieux M. (2012). MapDisto: fast and efficient computation of genetic linkage maps. *Mol. Breed.* 30 1231–1235. 10.1007/s11032-012-9706-y

[B25] Mathivathana M. K. Murukarthick J. Karthikeyan A. Jang W. Dhasarathan M. Jagadeeshselvam N. (2019). Detection of QTLs associated with mungbean yellow mosaic virus (MYMV) resistance using the interspecific cross of *Vigna radiata* X *Vigna umbellate*. *J. Appl. Genet.* 60 255–268. 10.1007/s13353-019-00506-x 31332718

[B26] Matsumura H. Hsiao M. C. Lin Y. P. Toyoda A. Taniai N. Tarora K. (2020). Long-read bitter gourd (*Momordica charantia*) genome and the genome architecture of nonclassic domestication. *Proc. Natl. Acad. Sci. U. S. A.* 117 14543–14551. 10.1073/pnas.1921016117 32461376 PMC7321978

[B27] Matsumura H. Miyagi N. Taniai N. Fukushima M. Tarora K. Shudo A. (2014). Mapping of the gynoecy in bitter gourd (*Momordica charantia*) using RAD-seq analysis. *PLoS One.* 9:e87138. 10.1371/journal.pone.0087138 24498029 PMC3907450

[B28] Matthew A. V. Mathew J. Malathi G. A. (1991). Whitefly transmitted disease of bitter gourd. *Indian Phytopath.* 44 497–499.

[B29] McKinney H. H. (1923). Influence of soil temperature and moisture on infection of wheat seedlings by *Helminthosporium sativum*. *J. Agric. Res.* 26 195–217.

[B30] Meng L. Li H. Zhang L. Wang J. (2015). QTL IciMapping: integrated software for genetic linkage map construction and quantitative trait locus mapping in biparental populations. *Crop. J.* 3 269–283. 10.1016/j.cj.2015.01.001

[B31] Meru G. McGregor C. (2016). Genotyping by sequencing for SNP discovery and genetic mapping of resistance to race 1 of *Fusarium oxysporum* in watermelon. *Sci. Hortic.* 209 31–40. 10.1016/j.scienta.2016.06.005

[B32] Miniraj N. Prasanna K. P. Peter K. V. (1993). “Bitter gourd *Momordica* spp,” in *Genetic improvement of vegetable plants*, eds Kalloo G. Bergh B. O. (Oxford: Pergamon Press), 239–246.

[B33] Montero-Pau J. Blanca J. Esteras C. Martínez-Pérez E. M. Gómez P. Monforte A. J. (2017). An SNP-based saturated genetic map and QTL analysis of fruit-related traits in zucchini using Genotyping-by-sequencing. *BMC Genomics* 18:94. 10.1186/s12864-016-3439-y 28100189 PMC5241963

[B34] Padidam M. Sawyer S. Fauquet C. M. (1999). Possible emergence of new geminiviruses by frequent recombination. *Virology* 265 218–225. 10.1006/viro.1999.0056 10600594

[B35] Picó B. Jose D. M. Nuez F. (1998). Evaluation of whitefly-mediated inoculation techniques to screen *Lycopersicon esculentum* and wild relatives for resistance to Tomato yellow leaf curl virus. *Euphytica* 101 259–271.

[B36] Polston J. E. Capobianco H. (2013). Transmitting Plant Viruses Using Whiteflies. *J. Vis. Exp.* 81:e4332. 10.3791/4332 24300175 PMC3984657

[B37] Raj S. K. Snehi S. K. Khan M. S. Rao G. P. (2010). First report of Pepper leaf curl Bangladesh virus strain associated with bitter gourd (*Momordica charantia* L.) yellow mosaic disease in India. *Australas. Plant. Dis. Notes* 5 14–16. 10.1071/dn10006

[B38] Rajinimala N. Rabindran R. Ramiah M. Kamlakhan A. (2005). Virus vector relationship of Bitter gourd yellow mosaic virus and whitefly Bemisia tabaci germ. Acta Phytopathol. *Entomol. Hung.* 40 23–30.

[B39] Rajinimala N. Rabindran R. (2007). First report on Indian cassava mosaic virus on bitter gourd (*Momordica charantia*) in Tamil Nadu. *India Australas. Plant. Dis. Notes.* 2 81–82. 10.1071/dn07033

[B40] Raman A. Lau C. (1996). Anti-diabetic properties and phytochemistry of *Momordica charantia* L. (Cucurbitaceae). *Phytomedicine* 2 349–362. 10.1016/s0944-7113(96)80080-8 23194773

[B41] Rao G. P. Behera T. K. Gaikwad A. B. Munshi A. D. Jat G. S. Krishnan B. (2018). Mapping and QTL analysis of gynoecy and earliness in bitter gourd (*Momordica charantia* L.) using genotyping-by-sequencing (GBS) technology. *Front. Plant Sci.* 9:1555. 10.3389/fpls.2018.01555 30429861 PMC6220052

[B42] Semiz A. Sen A. (2007). Antioxidant and chemoprotective properties of *Momordica charantia* L. (bitter melon) fruit extract. *Afr. J. Biotechnol.* 6 273–277.

[B43] Sharma S. Kang S. S. Sharma A. (2015). First report of mixed infection of Zucchini yellow mosaic virus and Tomato leaf curl New Delhi virus in bitter gourd in India. *J. Plant Pathol.* 97:2. 10.4454/JPP.V97I2.045 32896216

[B44] Tahir M. Haider M. S. (2005). First report of Tomato leaf curl New Delhi virus Infecting bitter gourd in Pakistan. *Plant Pathol.* 54:807. 10.1111/j.1365-3059.2005.01215.x

[B45] Takami K. Okubo H. Yamasaki S. Takeshita M. Takanami Y. (2006). A cucumber mosaic virus isolated from *M. charantia* L. *J. Gen. Plant Pathol.* 72 391–392. 10.1007/s10327-006-0301-0

[B46] Takeuchi S. Shimomoto Y. Ishikawa K. (2009). First report of Melon yellow spot virus infecting balsam pear (*Momordica charantia* L.) in Japan. *J. Gen. Plant Pathol.* 75 154–156. 10.1007/s10327-009-0143-7

[B47] Tiwari A. K. Sharma P. K. Khan M. S. Snhehi S. K. Raj S. Rao G. P. (2010). Molecular detection and identification of Tomato leaf curl New Delhi virus isolate causing yellow mosaic disease in Bitter gourd (*Momordica charantia*), a medicinally important plant in India. *Med. Plants* 2 117–123. 10.5958/j.0975-4261.2.2.018

[B48] Tokashiki I. Yasuda K. (1991). Diseases and pests of balsam pear (in Japanese). *Plant Prot.* 45 128–132.

[B49] Tomar S. P. S. Jitendra M. (2005). Severe mosaic caused by *Watermelon mosaic virus*-1 on bitter gourd (M. Charantia) in western U.P. J. Living. *World* 84:12.

[B50] Urasaki N. Takagi H. Natsume S. Uemura A. Taniai N. Miyagi N. (2017). Draft genome sequence of bitter gourd (*Momordica charantia*), a vegetable and medicinal plant in tropical and subtropical regions. *DNA Res.* 24 51–58. 28028039 10.1093/dnares/dsw047 PMC5381343

[B51] Voorrips R. E. (2002). MapChart: software for the graphical presentation of linkage maps and QTLs. *J. Hered.* 93 77–78. 10.1093/jhered/93.1.77 12011185

[B52] Wang Z. Xiang C. (2013). Genetic mapping of QTLs for horticulture traits in a F_2–3_ population of bitter gourd (*Momordica charantia* L.). *Euphytica* 193 235–250. 10.1007/s10681-013-0932-0

[B53] Warrier P. K. Nambiar V. P. K. Ramankutty C. (1997). *Indian Medicinal Plants. Vol 4.* Hyderabad: Orient Longman.

[B54] Wu S. Shamimuzzaman M. Sun H. Salse J. Sui X. Wilder A. (2017). The bottle gourd genome provides insights into Cucurbitaceae evolution and facilitates mapping of a Papaya ring-spot virus resistance locus. *Plant J.* 92 963–975. 10.1111/tpj.13722 28940759

[B55] Xu Y. Li P. Yang Z. Xu C. (2017). Genetic mapping of quantitative trait loci in crops. *Crop. J.* 5 175–184. 10.1016/j.cj.2016.06.003

[B56] Zhang G. Ren Y. Sun H. Guo S. Zhang F. Zhang J. (2015). A high-density genetic map for anchoring genome sequences and identifying QTLs associated with dwarf vine in pumpkin (*Cucurbita maxima* Duch.). *BMC Genomics* 16:1101. 10.1186/s12864-015-2312-8 26704908 PMC4690373

