# Supplementary material for: High-Density GBS-Based Genetic Linkage Map Construction and QTL Identification Associated With Yellow Mosaic Disease Resistance in Bitter Gourd (Momordica charantia L.)
Source: Front Plant Sci. 2021 Jun 24;12:671620. doi: 10.3389/fpls.2021.671620 (PMC8264296; doi:10.3389/fpls.2021.671620)
Supplement: Supplementary file 1 [file Data_Sheet_1.PDF]

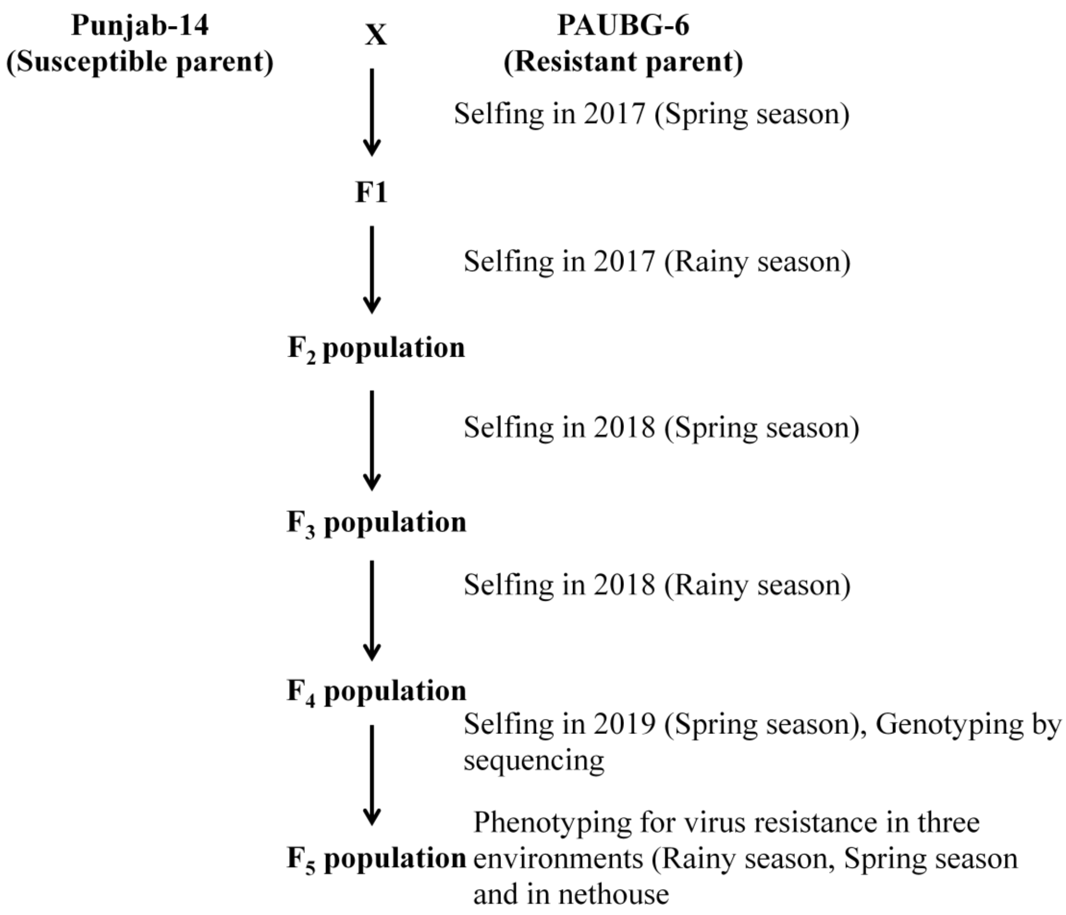

Figure S1 Schematic representation showing development of $F_{2:4}$ and $F_{2:5}$ lines used in the study

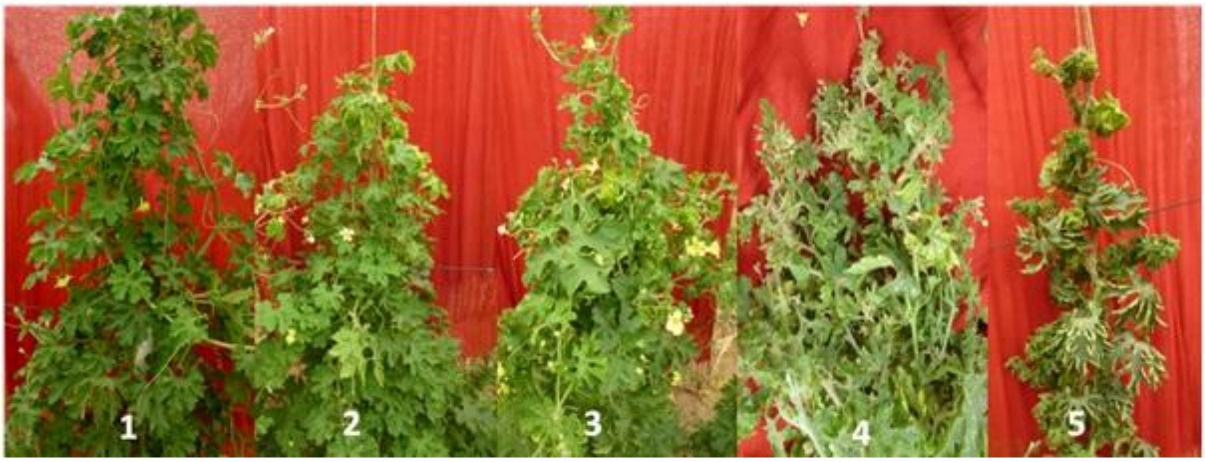

Figure S2 Disease grading during phenotypic evaluation of $F_{4:5}$ population

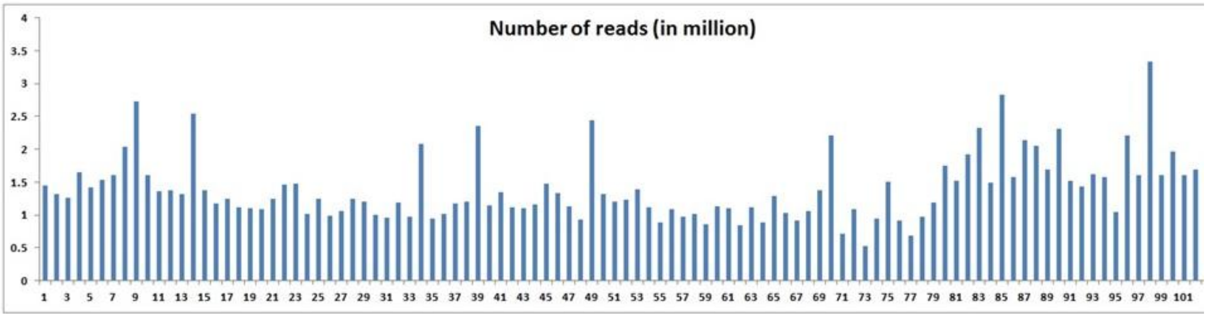

Figure S3 Number of reads generated per sample in F_4 individuals derived from the cross between Punjab-14 and PAUBG-6
