# Supplementary material for: High-Density GBS-Based Genetic Linkage Map Construction and QTL Identification Associated With Yellow Mosaic Disease Resistance in Bitter Gourd (Momordica charantia L.)
Source: Front Plant Sci. 2021 Jun 24;12:671620. doi: 10.3389/fpls.2021.671620 (PMC8264296; doi:10.3389/fpls.2021.671620)
Supplement: Supplementary file 2 [file Table_1.docx]

**Table S1** Scale used for scoring Yellow mosaic disease

| Score | Symptoms | Reaction |
| --- | --- | --- |
| 0 | No symptoms | Highly resistant |
| 1 | Upto 5% curling and clearing of upper leaves | Resistant |
| 2 | 6-25% curling, clearing of leaves and swelling of veins | Moderately resistant |
| 3 | 26-50% curling, puckering, yellowing of leaves and swelling of leaves | Moderately susceptible |
| 4 | 51-75% leaf curling, stunted plant growth and blistering of internodes | Susceptible |
| 5 | >75% curling, deformed small leaves, stunted plant growth with small flowers | Highly susceptible |
